# A Peculiar Case of Isolated Bilateral Tympanic Plate Fractures

**DOI:** 10.7759/cureus.55243

**Published:** 2024-02-29

**Authors:** Priyanka Bhat, Raman Sharma, J. C Passey, Niyati Kumari

**Affiliations:** 1 Otolaryngology - Head and Neck Surgery, World College of Medical Science and Research, Jhajjar, IND; 2 Otolaryngology - Head and Neck Surgery, Maulana Azad Medical College, New Delhi, IND

**Keywords:** nonsurgical treatment, tympano-mandibular joint, temporal bone injuries, mandible, external auditory canal stenosis, tympanic plate fractures

## Abstract

Tympanic plate fractures are uncommon injuries and carry the risk of external auditory canal stenosis. These injuries are often associated with fractures of adjacent bones like the mandible, maxilla, and temporal bone. Isolated bilateral tympanic bone fractures have rarely been reported. The most frequently advocated treatment for these injuries is surgical to prevent canal stenosis in the future. The effectiveness of non-operative management has been seldom reported. In the current case report, we present an uncommon injury with isolated bilateral tympanic plate fractures secondary to trauma to the mandible with no associated mandible or condylar fractures that were treated non-operatively. The functional outcomes were favorable at one year of follow-up.

## Introduction

The tympanic plate lies adjacent to the temporomandibular (TM) joint. The anterior and inferior walls of this external auditory canal (EAC) are formed by the tympanic portion of the temporal bone. The superior wall is formed by the squamous portion, and the posterior wall is formed by the mastoid portion of the temporal bone [1,2]. The bleeding from the external auditory canal can be due to injuries from the posterior auricular and superficial temporal branches of the external carotid supplying EAC and from the maxillary artery (the deep auricular branch and the anterior tympanic artery) [3]. 

Temporal bone fractures are divided into longitudinal or transverse fractures about the axis of the petrous portion of the temporal bone. Longitudinal fracture arises from a lateral blow to the temporal bone, and transverse fracture arises from force perpendicular to the long axis [3]. Any direct trauma to the chin may cause transmission of forces to the mandibular condyle and may cause injury to the tympano-mandibular joint. A bulge is produced in the anterior bony wall of EAC by the mandibular condyle and temporal bone’s glenoid fossa, which can limit visualization of the tympanic membrane [4]. With imaging techniques such as computed tomography (CT) scanning, the diagnosis of EAC fractures can be easily made [5]. High-resolution computed tomography (HRCT) temporal bone is the ideal investigation to diagnose the site and course of the fracture line, which may help us in prognostication and appropriate treatment. In a study by Altay et al. [1], out of 35 patients with isolated tympanic plate fractures, only four patients (11%) had bilateral tympanic plate fractures [1]. Complications of these fractures can include hearing loss and canal stenosis [6]. In the current case report, we present an uncommon injury with isolated bilateral tympanic plate fractures secondary to trauma to the mandible with no associated mandible or condylar fractures.

## Case presentation

**Figure 1:**
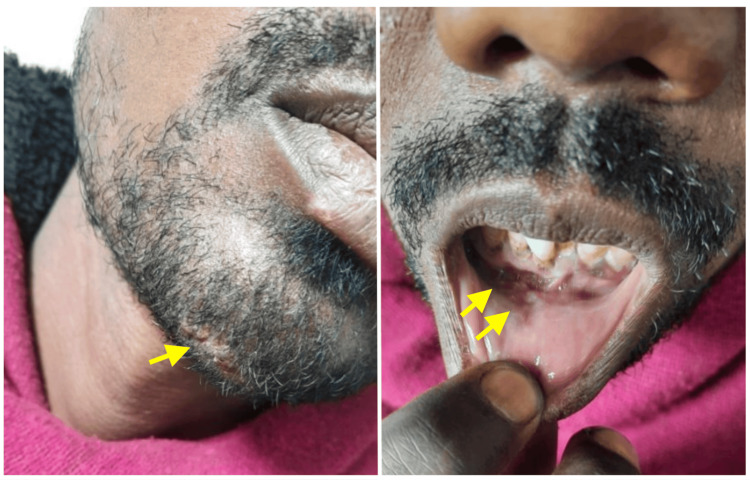
Figure showing the original site of impact resulting in laceration on the mentum in midline following trauma with an axe (single yellow arrow) and a intra-oral laceration (sutured) at lower gingivobuccal sulcus between two lateral incisors (two yellow arrows).

**Figure 2:**
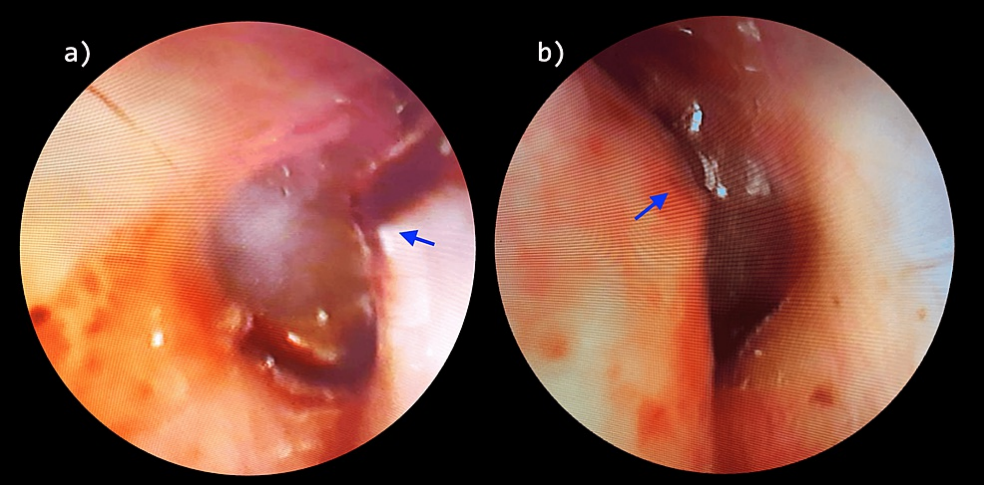
Figure showing oto-endoscopic images of right (a) and left (b) ears following the trauma to the mandible. The blue arrows show the bony projections on the anterior canal wall.

A 28-year-old male presented to the emergency ward with complaints of a wound over the chin and orally deep under the lower lip, and bleeding from bilateral ears following trauma to the chin with an axe. The patient complained of pain over the bilateral temporomandibular joint on jaw movement. Suturing was done over the chin (Figure 1), and on otoscopic examination, an anterior bulge of the external auditory canal with ear bleed from both ears (Figure 2) was observed. Rinne's Test was positive on both sides, and Weber's test was not lateralized.

**Figure 3:**
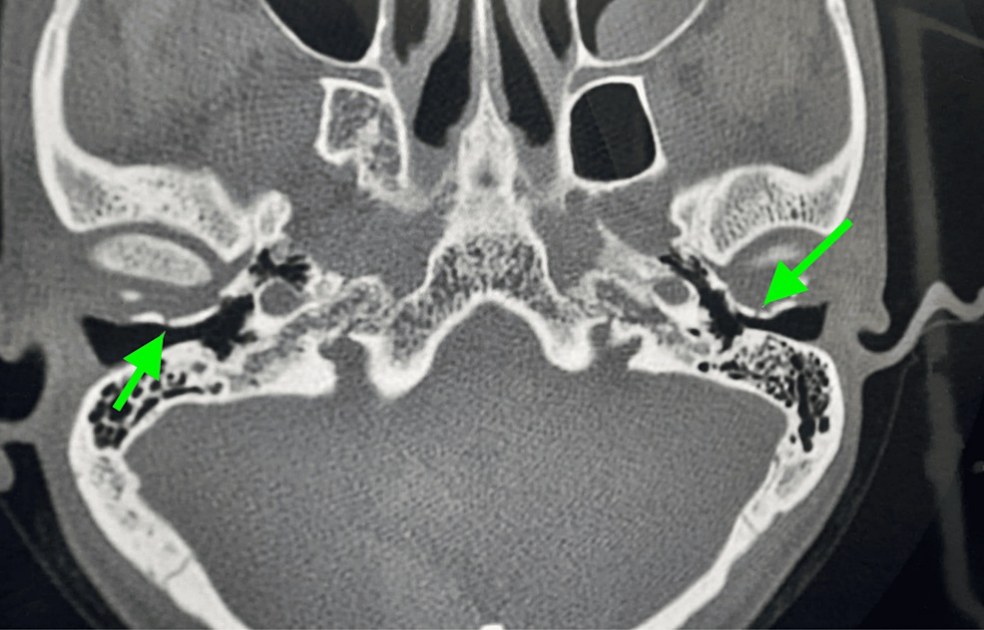
An HRCT axial cut showing bilateral undisplaced linear fractures (green arrows) of the anterior wall of the bony part of the bilateral external auditory canal (tympanic part). HRCT: high-resolution computed tomography

A high-resolution computed tomography (HRCT) was performed, which showed an undisplaced linear fracture of the anterior wall of the bony part of the bilateral EAC (tympanic part) with intact ossicular chain, tegmen tympani, and scutum (Figure 3).

The middle ear cavity, the mastoid antrum, the facial canal, and the inner ear were normal. The patient was followed up after one week for suture removal, and an oto-endoscopy was done, which showed an anterior bulge of the external auditory canal (Figure 2) and an intact tympanic membrane in both ears. Pure tone audiometry showed a normal hearing level in both ears. The patient was kept on conservative management and followed up for one year at two monthly intervals. In cases of tympanic ring fractures, complications such as repeated impaction of cerumen/repeated ear canal infections, etc are often encountered. Usually, canaloplasty is indicated to increase the cross-sectional area of the canal to avoid the above-mentioned complications [2,7,8]. One of the indications of surgery is a coexisting mandibular fracture. In our case, even after one year of follow-up, there were no signs of wax/debris collection in the canal and no conductive hearing loss despite persisting stenosis on otoscopic examination on follow-up visits that was similar to the initial examination. Pure tone audiometry was normal at each follow-up and at the end of one year.

## Discussion

Our case findings suggest that a conservative approach can help in favorable outcomes in bilateral tympanic plate fractures. The patient was followed for a year with normal hearing outcomes, and despite canal stenosis because of anterior bulge endoscopic examination gives a better visualization of the tympanic membrane. 

In 1977, Schmidseder et al. [9] found a 15% incidence of condylar fractures with EAC fractures. When no fracture or displacement of the condyle was found, it was hypothesized that an isolated anterior-wall EAC laceration was due to posterior displacement of the condylar head [10]. The temporal bone fracture results in complications such as hearing loss, cranial neuropathy or dysfunction, cerebrospinal fluid (CSF) leak, and vestibulopathy [5]. Dahiya et al. [11] reported a higher incidence of facial nerve damage, CSF leaks, profound hearing loss, and frequent intracranial complications with the involvement of the otic capsule compared with those that spared the bony labyrinth. In our case, fortunately, there were no complications like tympanic membrane injury, ossicular dislocation, or associated mandible fracture leading to malocclusion of teeth and difficulty chewing.

Gomes et al. [12] documented a tympanic plate fracture due to posterior dislocation of the mandibular condyle. This case adds to the 13 articles in the literature that jointly report 24 fractures of the tympanic plate following mandibular trauma. In 11 cases, the tympanic plate fracture was present on the right side; in nine cases, the fracture was on the left side, and in four cases, it was bilateral [12]. Wood et al. [5] reported tympanic plate fractures associated with ossicular disruption and scutum fractures with temporal bone fractures. We did not evaluate the relationship between ossicular disruption and scutum fracture associated with tympanic plate fractures. Bleeding from the EAC is the main symptom in patients with fractures of the tympanic plate. Laceration of the anterior EAC wall, tympanic membrane rupture, injury of the middle ear ossicles, and hemotympanum can also be seen [1]. We observed a bulge in the bilateral anterior wall of EAC associated with bilateral hemotympanum and bleeding from EAC with an intact tympanic membrane. Hearing loss and canal stenosis are common following EAC injuries, but this was not valid in our case, wherein there was no hearing loss. While canal stenosis was there, we didn't consider the operative management as the patient didn't have any hearing loss or cerumen impaction. We felt that canaloplasty with a thin anterior fractured wall of the external auditory canal may expose the TM joint capsule and would risk infection and restenosis.

The most common causes of injury are accidents, falls from height, mandibular trauma, and physical assault. De Andrade et al. [13] reported patients with mandibular trauma. In their study, the most frequent causes were vehicular accidents (48.8%), falls (26.5%), and physical assault (23.5%). Isolated mandible fractures predominated, but there was an association with fractures of other bones [13]. 

## Conclusions

Isolated bilateral tympanic bone fractures without associated mandible, maxillary, or condylar fractures are uncommon injuries. While surgical management has been advocated in literature to avoid EAC stenosis, the role of non-operative treatment has been sparsely investigated. A conservative non-operative approach with regular follow-up may be considered in the absence of mandibular fractures, otologic damage like ossicular or tympanic membrane disruption, complete canal stenosis, and conductive hearing loss. Further, long follow-up studies are needed to better characterize the outcomes of such an approach in isolated bilateral tympanic bone fractures.
